# Mapping the consumer foodshed of the Kampala city region shows the importance of urban agriculture

**DOI:** 10.1038/s42949-023-00093-1

**Published:** 2023-03-02

**Authors:** Lisa-Marie Hemerijckx, Gloria Nsangi Nakyagaba, Hakimu Sseviiri, Katarzyna Janusz, Michelle Eichinger, Shuaib Lwasa, Julian May, Peter H. Verburg, Anton Van Rompaey

**Affiliations:** 1 Department of Earth and Environmental Sciences, KU Leuven, Leuven, Belgium; 2 Fonds Wetenschappelijk Onderzoek (FWO) Vlaanderen, Brussel, Belgium; 3 Urban Action Lab (UAL), Department of Geography, Geo-Informatics and Climatic Sciences, Makerere University, Kampala, Uganda; 4 Department of Geography and Environmental Sustainability, University of Oklahoma, Norman, USA; 5 Institute for Environmental Studies (IVM), Vrije Universiteit Amsterdam, Amsterdam, The Netherlands; 6 International Institute of Social Studies (ISS), Erasmus University Rotterdam, Rotterdam, The Netherlands; 7 DSI-NRF Centre of Excellence in Food Security, University of the Western Cape, Cape Town, South Africa

**Keywords:** Sustainability, Development studies, Geography

## Abstract

Due to rapid urbanisation, food systems in sub-Saharan African cities are increasingly under pressure. Through the lens of a foodshed, this paper quantitatively analyses the spatial extent of the food provisioning area for consumers of different socio-economic status in Kampala (Uganda). Based on a primary dataset of surveys with households and food vendors, we map the foodshed by registering where consumers obtain their food, and the origin of where it is grown. We show that 50% of the food consumed in the city originates from within a 120 km proximity to Kampala, including 10% from within the city itself. At present, urban agricultural activities are twice as important as international imports for the urban food provision. Established, high-income urban dwellers have a more local foodshed due to their broad participation in urban agriculture, while low-income newcomers rely heavily on retailers who source food from rural Uganda.

## Introduction

Sub-Saharan Africa (SSA) is facing a double issue of very rapid urbanisation paired with high levels of food insecurity exacerbated even more by recent global shocks such as Covid-19 or the Russian-Ukrainian war^1^. With rapid urban growth and socio-economic change in SSA, food consumption and production patterns are transforming, and with that, the length of supply chains^2,3^. On the consumption side, there is a general food demand increase due to urban population growth. In addition, urban dwellers of middle- to high-income groups are changing their diets towards more processed (fast) foods and more animal-based proteins^4^, which are often associated with high social status^5^. On the production side, when urban growth is spatially translated to horizontal sprawl, uncontrolled urbanisation can lead to loss of croplands in the urban and peri-urban zone, directly impacting the regional food system^6^.

With urban agricultural practices being very common across SSA, the question remains to what extent urban food provision of the future will be affected if more and more patches of prime (informal) agricultural land have to make room for built-up area^7^. There is a longstanding discussion among scholars about the importance of urban agricultural activities to the urban food system. Studies on the limited reach of urban agriculture argue that it is at present scarcely contributing to the urban foodshed, with a limited potential to contribute to food security^8,9^. In addition, concerns have been raised regarding the food quality and safety of products sourced in SSA’s urban areas^10^. On the other hand, food systems researchers argue that while the evidence base is weak, there are signs that urban agriculture has a positive association with consumer’s dietary diversity and food consumption^11^. Beyond poverty alleviation, by lowering transport costs and emissions and reducing urban heat island effects, (peri-) urban agriculture can help mitigate and adapt to the unavoidable impacts of climate change, which could disrupt future food production^10^. Scholars also stress the potential importance of urban agriculture for urban dweller’s access to land, even if only a small portion of the city’s food supply is sourced from within the city^12^. Hemerijckx et al. (2022) have shown that mainly socio-economically established urban dwellers have access to urban agricultural land, while recent rural-urban migrants cannot depend upon urban agriculture for their food provisioning^13^. What both arguments for and against a strong dependency on urban agriculture lack are quantifiable and comparable statistics on the quality and quantity of the food sourced in metropolitan areas. Information combining data across the entire food system, from (urban) farm to consumer, can be crucial for city managers who aim to design strategies for more secure, just, transparent and sustainable African urban food systems^14^.

Authorities often view food systems as an agricultural mandate, framing it as a (rural) production problem^15^, and have therefore been slow to incorporate food systems in urban planning policies^3^. Nonetheless, food demand and production are not the only challenge facing Africa’s rapidly urbanising food systems; food accessibility is a fundamental issue as well^13^. Most food-insecure households are limited by physical, social and economic access to food^16^. Consumer’s food accessibility is affected by dynamics of poverty and socio-economic segregation^13,17^, proximity to food sources^18^, price volatility, healthy food availability, market disruption and loss of farmland in favour of other land uses^19^. These drivers, including their interactions, need to be considered in policies addressing city food insecurity and injustice. The food demand and accessibility in urban areas strongly shape the local food retail system, the peri-urban and rural hinterlands producing the bulk of agricultural products^20^ and even the distant areas through tele-coupled global markets^21^. Thus, as SSA’s urban food systems transform, new challenges and opportunities arise for consumers, farmers and enterprises in the food value chain.

Current literature on urban food systems focuses on the city region food systems approach^22–24^, which is used as a holistic policy framework that includes urban, peri-urban and rural landscapes. This study analyses the city region food system through the lens of a so-called foodshed. Analogous to a watershed, a foodshed can be described as: “*the geographic area from which a population derives its food supply*”^25^ (Peters et al., 2008, p. 2). Early foodshed studies advocated for highly localised food systems and high levels of urban food self-sufficiency (e.g.,^26–28^). The advantages of local foodsheds are that they can improve consumer-producer relationships, decrease transport costs, greenhouse gas emissions and reliance on (inter-) national infrastructure, and may improve the economic viability of local communities^25,28^. While shocks such as international conflict, inflation, fuel shortages or transport strikes may be mitigated by local foodsheds^27^, high self-sufficiency levels can also pose risks in terms of local civil unrest or natural hazards^1^. Diversified foodsheds might alleviate the risks, which is why a balance between the local, regional, international and global scales is increasingly presented as the solution to mitigate these vulnerabilities^10,29,30^. Karg et al. (2016) argue that the pathway towards resilient urban food systems in SSA could be to enlarge the foodshed by diversifying food supply in terms of both foodstuffs and their locations. More empirical research on this question is needed, especially since the effects of urban sprawl on this system have generally been neglected.

There is currently a lack of empirical studies and methodologies for mapping and quantifying urban consumers’ foodsheds, especially in the rapidly urbanising SSA region. Scholars generally analyse foodsheds either in hypothetical terms, as the area that would potentially be capable to feed a city’s population, or in terms of the food flow, i.e., the geographies of the food value chain, or as a combination thereof^31^. Potential capacity studies are a growing body of literature mainly in North American case studies (e.g.,^32–34^), in some Western European case studies (e.g.,^35,36^) and in global-scale optimisation model approaches (e.g.,^29^). A very limited number of studies have spatially mapped the empirical foodsheds or food flows towards urban areas. While a first food flows study was conducted in 2007 by Drechsel et al.^4^, to our knowledge, the only recent empirical example from SSA is by Karg et al.^24^ and Karg et al.^2^. They quantitatively mapped the foodsheds for four West African cities for selected crops. However, their methodology is from a supply standpoint, as they counted the number of foodstuffs being transported into these cities (mainly by road). As not all actors in the food system are included, this leaves one to wonder which vendor types sell the food, and who (dwellers of which socio-economic groups) will be consuming it. The complex dynamics of geographical contexts, coupled with the variation in agro-ecological productivity, imply that there are multiple pathways to realising sustainable urban food systems that include local or regional supply chains^35^. Understanding the shape and size of the foodshed can thus help urban planners and consumers to work towards a more resilient, sustainable and food-secure future^25,31^.

In short, urban sprawl in SSA has transformed city food systems in the last decades. Planning authorities have historically not been able to adequately cope with changing urban food supply, since the actual food flows are undocumented for most cities. This is largely due to the (semi-) informal nature of African urban food systems^37,38^. Hence, there is a need for empirical studies and methodologies on how to quantify and visualise the urban food system, including informal vendors and urban agricultural activities. Information on food consumption and accessibility patterns is also scarce^13^, which is why both food supply to and consumption by urban dwellers of different socio-economic groups should be quantified and visualised. This information can then be used as a decision support tool for urban planners, who increasingly wish to incorporate (informal) urban food systems in their policies^39,40^.

**Fig. 1:**
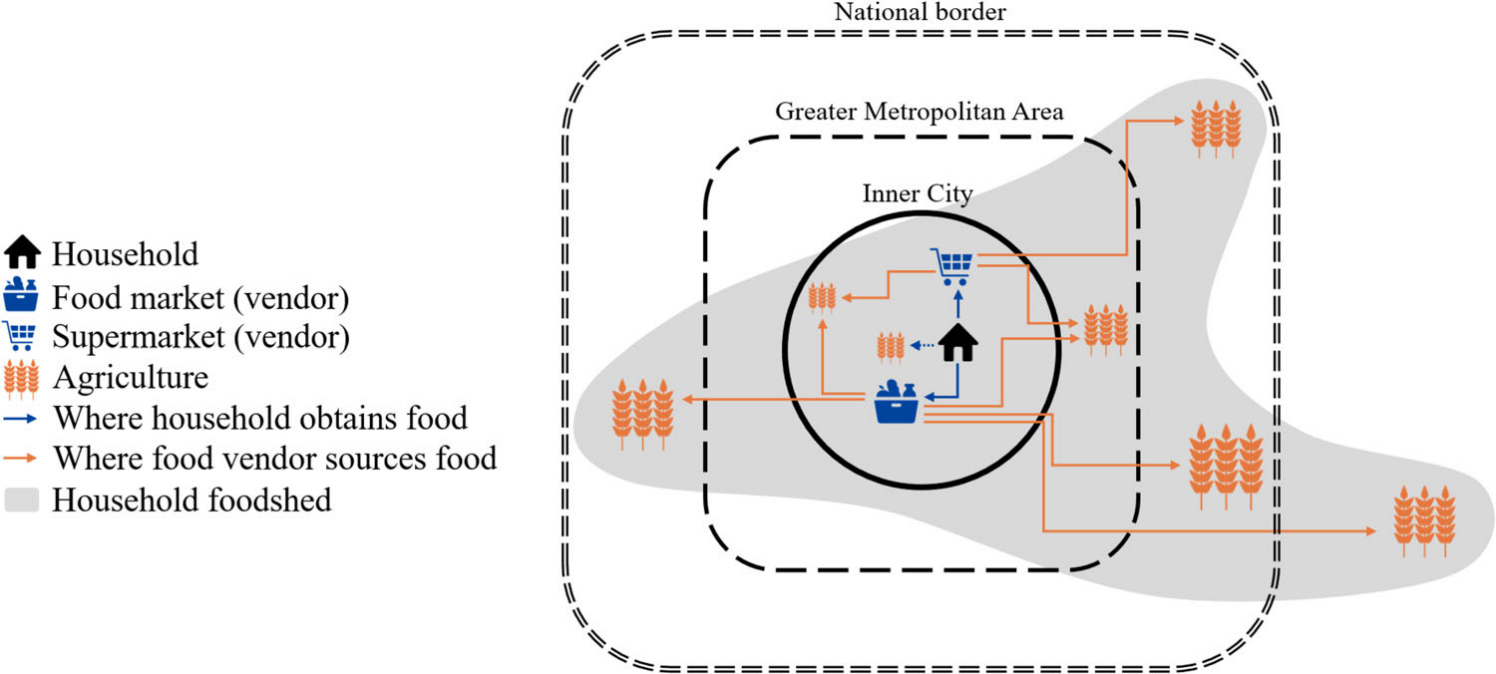
Schematic representation of the foodshed of an urban household. The household indicates where their food supply is obtained. The vendors indicated by the household indicate the agricultural locations where their food supply is sourced (generally via suppliers). Households and vendors may also source food directly from the farm. The area encompassing this entire system is the household’s foodshed.

The aims of the present study are (i) to trace the locations of the food sources for households of different socio-economic groups and for different food vendor types and (ii) to empirically map the foodshed of urban consumers, thereby uncovering the spatial extent of the food provisioning area. The present study aims to add nuance to the ongoing polarised debate about globalised versus localised foodsheds, by including the perspective of urban consumers of different socio-economic status. Figure 1 demonstrates our approach on tracing the food flow starting from the household consumer, via the (formal or informal) vendors where the household obtains their food supply, to the origins of the food. The link ‘Where food vendor sources food’ is usually not a direct one: food vendors generally obtain their produce via suppliers, rather than directly from the farm. Details on the practical implementation of this research design can be found in the ‘Methods’ section. This research aims to quantify and delineate the urban foodshed, demonstrating the relative contribution of various geographical scales, including the city region. In order to delineate foodsheds at different scales and for various foodstuffs, one of three units must be chosen to define various supply regions: by weight, by nutritional value (e.g., kcal), or by (equivalent) retail value.

**Fig. 2:**
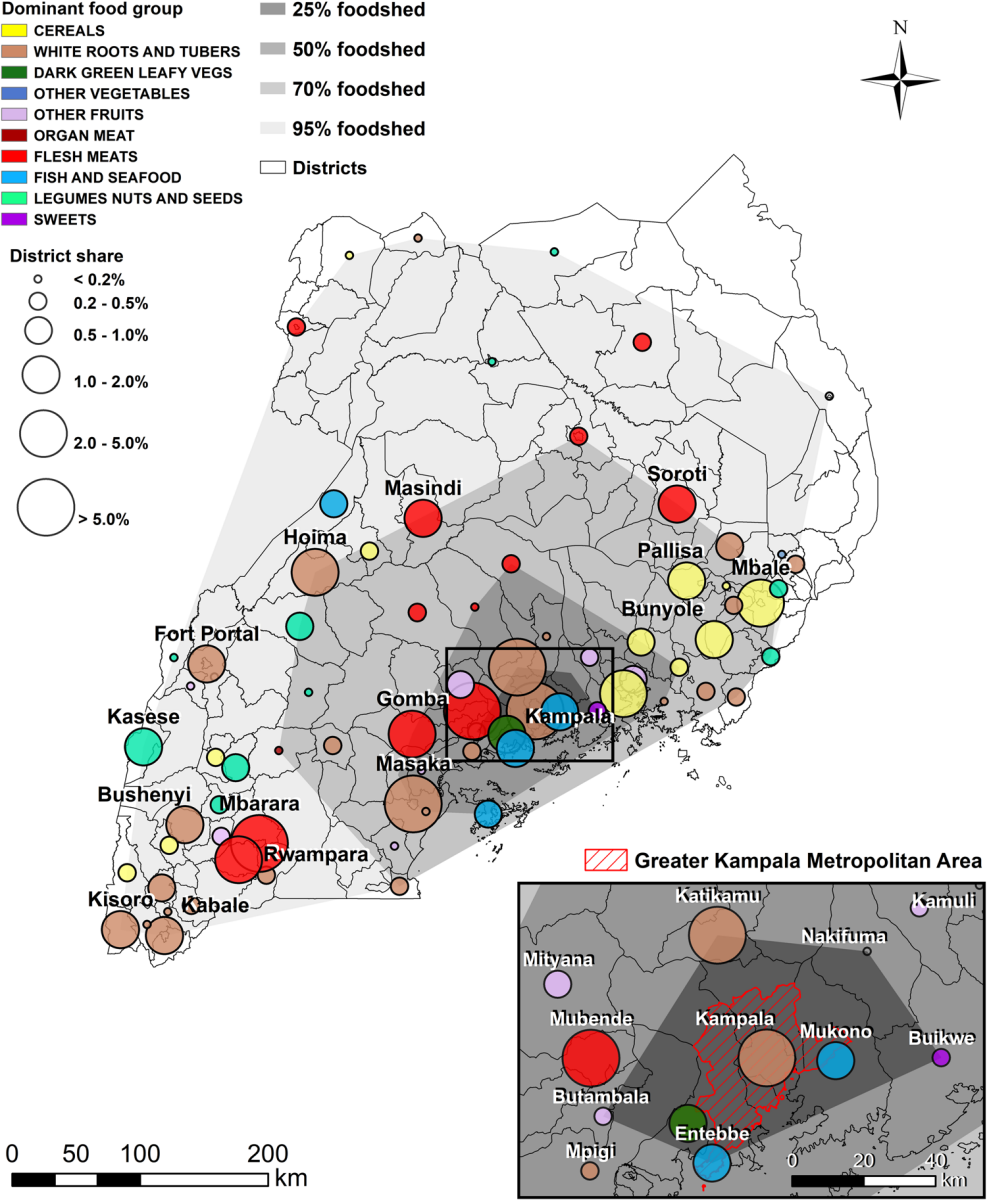
Foodshed of consumers in Kampala. Polygons delineate the percentage of food that is provisioned within that area. The dots represent district share of the total foodshed (in terms of UGX/day). The dominant food group originating from each district (based on vendor data) is also shown. Food groups not shown in the legend were not dominant in any district.

The Greater Kampala Metropolitan Area (GKMA, Fig. 2) in Uganda is chosen as a case study to analyse the urban foodshed. The city region is considered to be representative of many sprawling urban centres in SSA, as it has a population exceeding 4 million people, which is growing by over 5% yearly^41,42^. This rapid, uncontrolled urban growth is due to high fertility rates combined with employment-driven rural-urban migration^43^ and poorly defined property rights^44^, resulting in agricultural land encroachment. Moreover, the GKMA has a high dependence on urban agricultural activities for its food provision^12^. In this study, we refer to any agricultural activities taking place within the boundaries of the GKMA as ‘urban agriculture’. Vermeiren et al. (2012) estimated that in 2010, almost one-third of the surface of the GKMA was used as agricultural land. In Kampala, urban agriculture is often practiced formally or informally on communal land, or on one’s own backyard space. The practice was illegal until 2006^45^ as it was considered economically insignificant and a health risk^12^. As a result, (peri-) urban agricultural activities generally take place on medium- to very small-scale plots located amidst built-up land. The dominant urban agricultural activity is crop growing in open air^7^.

Hemerijckx et al. (2020) have defined four socio-economic clusters (SECs) of households living in Kampala: ‘established high income’, ‘established low income’, ‘newcomers middle income’ and ‘newcomers low income’^46^. Socio-economic clusters are defined relative to the other clusters in our dataset only. A summary of the key socio-economic variables defining these SECs can be found in Supplementary Table 1. The established, high income households have generally been living in Kampala for over two decades and have a daily food expenditure per person of over 4600 Ugandan Shillings (UGX), corresponding to 1.20 USD. It is therefore worth noting that the majority of the relatively ‘established high income’ households in this dataset should still be considered part of the rising (upper) middle class in sub-Saharan Africa^47^. This SEC has the highest levels of dietary diversity and the lowest prevalence (< 5%) of severe food insecurity according to the Food Insecurity Experience Scale^13,48^. On the other side of the socio-economic spectrum are the newcomers who have generally lived in Kampala less than 10 years, with a comparatively low household income and food expenditure of around 3000 UGX. Over one in five households of this SEC can be considered severely food insecure and they mostly reside in small homes or slum dwellings^46^. Over 60% of all established dwellers, irrespective of income, are actively engaging in urban agricultural activities, while for newcomers this value is under 25%. These four SECs will be used to analyse food access sources and to display the foodshed graphs.

## Results and discussion

### Food sources

Table 1 shows where the clustered households obtain the five food items they consume the most, in terms of Ugandan shillings (UGX) spent at each source. Because some households mentioned fewer than five types of food, the average respondent across all SECs provided this information for 4.67 food items (total *n* = 3490). These will further be referred to as the staple foods of the household. In total, 63 unique food types were mentioned as staple foods by households, the most popular of which were rice (*n* = 545, categorised as ‘cereals’), matoke bananas (*n* = 520, categorised as ‘white roots and tubers’) and beans (*n* = 473, categorised as ‘legumes, nuts and seeds’).

**Table 1:** Food sources where surveyed households (*n* = 747) of different SECs obtain their five staple foods.

Food source	Established high income *n* = 221	Established low income *n* = 134	Newcomers middle income *n* = 177	Newcomers low income *n* = 215	Dataset *n* = 747
Home grown by household	15.7%	7.9%	1.9%	1.0%	7.4%
Family, friends or neighbours	1.2%	0.5%	1.4%	0.9%	1.1%
Mobile street vendor	4.2%	1.8%	2.9%	0.8%	2.5%
Fixed street vendor	20.2%	20.7%	24.2%	17.2%	20.0%
Market vendor	29.4%	33.4%	33.5%	14.8%	26.0%
Retailer	27.6%	35.4%	34.2%	65.1%	42.0%
Supermarket	1.6%	0.4%	1.9%	0.1%	1.0%

Percentage in terms of UGX/person/day spent at each source or equivalent (when home grown or gifted).

All SECs obtain their staple food mostly from retailers (average 42.0%), market vendors (average 26.0%) and fixed street vendors (average 20.0%). Consistent with previous research on household food accessibility in Maputo, Mozambique^49^, more than three quarters (76.2%) of the staple food items consumed by surveyed households are obtained within 30 min walking distance. While supermarkets provide the smallest share of the household staple food source (dataset average 1.0%), middle- to high-income clusters visit supermarkets slightly more with 1.9% and 1.6% of their monetary value being spent there on staple foods. Lower-income households hardly ever visit this vendor type. Our findings on the use of different retail outlets confirm the results of Wanyama et al.^50^ who show that supermarkets account for only 0.4% of food expenditure of slum dwellers in Kampala, while retailers, local markets and roadside vendors are the most popular retail outlets respectively. Thus, concerns regarding the ‘supermarketisation’ of the urban food systems in SSA may be overstated, at least for low-income consumers in countries with limited geographical proximity (and associated economic ties) to South Africa, where most supermarket chains are based^51^.

On average, 7.4% of the value of household staple foods is produced by the household themselves. ‘Home grown by household’ may also imply food produced on land outside of the GKMA, meaning this is not always classified as (peri-)urban agricultural activity. This statistic is validated by UBOS, who report 5.4% own production for Kampala in 2018/2019^52^. Typically, the established urban dwellers with a higher income produce significant portions (15.7%) of their own staple food, as they often have access to farmland and the resources to cultivate it^46^. The established high income households have the most diversified food source pattern. The reason for this is twofold: partly due to their high involvement in urban agriculture, and partly because their income level allows for a more optimal resource allocation. This tactic of diversifying household food sources, adopted mainly by high- to middle-income urban dwellers, has previously been observed in other SSA cities^17,49,53^. The new rural-urban migrants with a low income are the most dependent on a single food source, with 65.1% of their food supply originating from retailers. Retailers are likely to offer credit arrangements, especially to households with whom they have a good relationship, which might be why the lower income SECs gravitate towards this vendor type^17,54^.

Following the approach depicted in Fig. 1, we trace the food from urban vendors towards its source. Table 2 shows how the surveyed food vendors obtain the food they sell in terms of UGX/day (retail value) spent at each source. Vendors could indicate up to 10 foodstuffs, but on average provided information on 3.30 food items (total n = 1001). In total, we collected information on 85 unique food types sold by vendors in Kampala. Table 2 shows that most food vendors obtain their produce via middlemen (42.7%) or at another market (35.1%). We define the middlemen as local (usually self-employed) merchants who purchase food in bulk and transport it from farms or markets in rural or peri-urban areas towards food vendors in the inner city. Less than a fifth (18.6%) of the produce supply by vendors is obtained directly from the farm. In addition, most vendors do not sell any food they themselves cultivated, i.e., most food vendors are not also farmers, with the exception of some fixed street vendors (3.6%) and retailers (7.8%). Retailers are also the only vendor type in our dataset who sell food that was obtained via family, friends or neighbours (6%). Mobile street vendors are heavily dependent on other food markets (86.6%), while surprisingly, supermarkets and wholesalers rely strongly on middlemen (70.2% and 83.6% respectively).

**Table 2:** Food sources where the surveyed food vendors (*n* = 303) obtain their supply.

Food source	Mobile street vendor *n* = 16	Fixed street vendor *n* = 112	Market vendor *n* = 112	Retailer *n* = 57	Supermarket *n* = 3	Wholesaler *n* = 3	Dataset *n* = 303
Home grown by vendor	0.0%	3.6%	0.1%	7.8%	0.0%	0.0%	2.5%
Family, friends or neighbours	0.0%	0.0%	0.0%	6.0%	0.0%	0.0%	1.1%
Farm	0.0%	21.0%	23.0%	8.1%	0.0%	16.4%	18.6%
Middleman	13.4%	31.4%	49.6%	40.9%	73.5%	83.6%	42.7%
Other food market	86.6%	43.9%	27.3%	37.2%	26.5%	0.0%	35.1%

Percentage in terms of UGX/day equivalent retail value spent at each source.

According to our survey, retailers have the most diversified food source pattern. The prevalence of middlemen and farm-to-market strategies implies that Kampala’s urban foodscape is at present not corporate dominated. This semi-informality is observed in other SSA cities as well, such as in Dar es Salaam, Tanzania^37^ or Tamale, Ghana and Ouagadougou, Burkina Faso^38^. The limited influence of supermarkets in the urban foodscape is in stark contrast to trends that have emerged in South Africa and in some middle-income SSA countries over the past two decades^55^. Our findings indicate that, similar to urban food systems in Ethiopia and Tanzania, Kampala’s food system is in a transitional stage^56^, in-between a traditional and a modern stage, as it heavily relies on middlemen who transport food from rural to urban areas.

### Foodshed

Food flows are traced from household consumers of different SECs, to food vendors, to the origins of the staple foods. We use the equivalent retail value in UGX to quantify the foodshed. The resulting map, based on household staple foods, is shown in Fig. 2. The foodshed of Kampala is relatively confined, with over 95% of the food provisioning originating from within Uganda’s borders. Our findings correspond closely to the national self-sufficiency ratio of 95.4% reported by UBOS^57^ and the caloric self-sufficiency ratio of 93% reported by FAO^58^. The 4.2% that is not mapped consists of imports from mainly Tanzania (2.1%), Pakistan (0.8%), Kenya (0.4%) and Rwanda (0.3%). Uganda’s limited import dependency is characteristic to the food systems in the SSA region, with all the neighbouring countries’ caloric self-sufficiency ratios being around 90% with the exception of Kenya (54%)^58^.

Almost one tenth (9.8%) of the food retail value consumed by surveyed households in Kampala is grown within the GKMA, and 25% can be found within 50 km from the inner city. This implies that urban agricultural activities are currently twice as important as international imports for the city’s food provision. The 50% city region foodshed can be found within little over a 100 km radius from Kampala. Despite methodological differences, the spatial extent (and specifically, the 50% threshold size) of Kampala’s city region food system closely correlates to previous urban foodshed studies carried out in Tamale, Ghana and Ouagadougou, Burkina Faso^24^. The 100 km radius appears to be a significant spatial benchmark in city region food system or foodshed mapping exercises^29,36^. While this might seem a purely symbolic distance, for example because some Northern American studies adhere to a distance of 100 miles instead^33^, we theorize that the 100 km radius is a recurring geographical measure because in most locations around the world, this distance can be travelled by road in a return trip during the duration of one work day. Future urban foodshed studies should pay close attention to this geographical scale as it sets a common measure when analysing the spatial extent of food provisioning areas.

Supplementary Table 2 shows how newcomer clusters spend most of their food budget on ‘cereals’ (mainly rice), while established clusters spend most on ‘white roots and tubers’ (mainly matoke bananas). ‘Flesh meats’ come in second place for the high- to middle income urban inhabitants, and are less important for low-income consumers. Because of its relatively high retail value, ‘flesh meats’ result as the dominating food group for multiple districts across Uganda. ‘White roots and tubers’ are the dominant food group grown in Kampala and Katikamu (Luweero), as well as in many south-western and central-eastern districts. ‘Fish and seafood’ logically dominate only in districts adjacent to Lake Victoria and Lake Albert. There is an evident provisioning area of ‘cereals’ located in the east of Uganda. Production in multiple districts adjacent to Kampala (Kayunga, Jinja, Mityana) is dominated by ‘other fruits’. The dominant food groups originating from each district are comparable to the ‘livelihood zones’ mapped for Uganda by USAID (2013). The livelihood patterns closest to Kampala focus on production of maize (‘cereals’), banana (‘white roots and tubers’), cattle (‘flesh meats’) and fishing^59^. The main difference between the foodshed map in Fig. 2 and the livelihood zones map is the prevalence of coffee-based livelihoods, one of the major cash crops^60^, which is not a staple food for most consumers in our household survey.

**Fig. 3:**
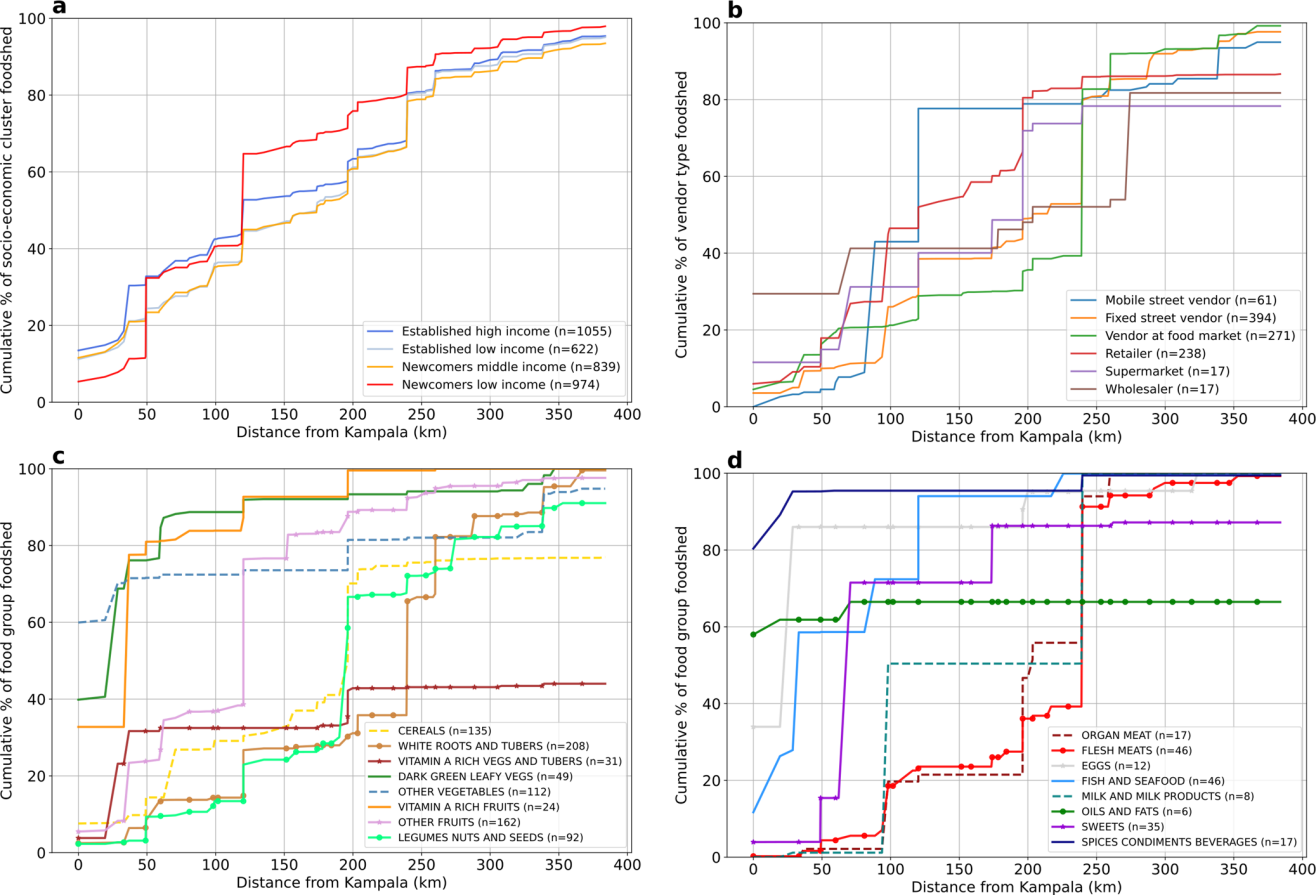
Cumulative contribution to the foodshed by distance from Kampala. **a** Shows the cumulative distance from Kampala of the foodshed for different socio-economic groups of consumers in Kampala. **b** Shows the cumulative distance from Kampala of the foodshed for different vendor types. **c** Shows the cumulative distance from Kampala of the foodshed for the 8 plant-based food groups defined by FAO (2010). **d** Shows the cumulative distance from Kampala of the foodshed for the 8 non-plant-based food groups defined by FAO (2010). Values for n shown in legend are based on the number of food items included in each analysis.

Figure 3 shows the cumulative distance from the city centre of Kampala for the foodshed of the various (a) SECs, (b) vendor types and (c and d) food groups. Food origin location(s) within the GKMA are considered to be at a distance of 0 km. Figure 3 a shows the established high-income households have the most local consumption, with 12.7% of their food supply sourced within the GKMA. While for most SECs the cumulative foodshed contribution steadily rises with distance from the city, the low-income newcomers have the most convex curve (starting at only 5.2% of their foodshed sourced within Kampala). Considering the significant differences in the food security and dietary diversity of the different SECs (Supplementary Table 1), and in their overall food consumption patterns (Supplementary Table 2), is it striking that the differences in the cumulative size of the foodshed are limited. Supplementary Fig. 1 demonstrates the shape of the 50% foodshed for each SEC. The more local consumption of the established high-income households is shown clearly, with a very small polygon delineating the 25% foodshed for this SEC. The ‘newcomers low income’ are the only SEC where the 70% foodshed can be mapped at less than 200 km from Kampala. While for established clusters, ‘white roots and tubers’ dominate local production, for newcomers the ‘cereals’ are the dominant food group originating from within Kampala. This is in line with the observations in Supplementary Table 2, showing that the main staple carbohydrate for established urban dwellers are ‘white roots and tubers’ while, for newcomers, this is ‘cereals’. Although diets based on tubers require a smaller area for cultivation than cereal-based diets^4^, the total foodshed size is similar for all SECs. Despite the fact that the diets of high-income households are more diverse (Supplementary Table 2), this does not directly imply that wealthier households have a larger foodshed for the case study of Kampala.

Food vendors (Fig. 3b) show a larger variety in their foodshed distribution. Mobile street vendors portray the most convex curve, while vendors at food markets source their produce further away. This is most likely due to the fact that mobile street vendors mainly sell ‘other fruits’ (41.5% of the retail value they have in stock) and ‘fish and seafood’ (31.6%) (Supplementary Table 2), while market vendors sell mainly ‘white roots and tubers’ (40.8%) and ‘flesh meats’ (36.2%). Fixed street vendors, similar to market vendors, focus most on ‘flesh meats’ (49.4%) and ‘white roots and tubers’ (19.4%). Retailers, on the other hand, sell mainly ‘cereals’ (47.0%), ‘legumes nuts and seeds’ (20.3%), as well as dairy products (15.0%) (Supplementary Table 2). As shown in Fig. 3c, about 30% of cereals are imported (this mainly involves rice from Pakistan), this explains why over 10% of Kampala’s retailer foodshed value originates from abroad. Although it is not surprising that supermarkets contain the most international foodshed, due to the limited sample size for supermarkets and wholesalers (*n* = 3 for both vendor types) and their minor contribution to the consumer foodshed, we will not further discuss their foodshed shape.

We identify three ongoing developments that will likely impact the shape and size of the urban foodshed. First, with continued rapid urban sprawl, it is likely that the foodshed of Kampala will change in shape and size. As the city grows, either horizontally or via ‘infilling’^41^, (peri-) urban agricultural land will make way for built-up area and the local foodshed will have to become larger. This development will mainly have a significant impact on the food provisioning of the more established urban dwellers, who might lose out on their current advantage regarding urban agriculture. Second, climate change is increasingly causing droughts and floods, as well as pests and diseases which impact agricultural productivity across SSA. As the food system in the Kampala city region relies so heavily on food from within national borders, the effects of climate change will make Kampala’s foodshed (and that of many other SSA cities) increasingly vulnerable^10^. Third, if dietary change towards more animal protein continues, socio-culturally linked to social status and prestige^5^, this will further impact Kampala’s future foodshed.

The effects of these three developments are already taking place and are intertwined: while urban agriculture is the most flexible food source for households, used as an insurance policy for challenging times^12^, its existence is being threatened by both urbanisation and climate change related disasters. In addition, an increased animal protein consumption will either require more imports, or more land to be allocated for livestock. A geographically larger foodshed will go hand in hand with increased transportation costs and emissions^28,61^. However, a larger foodshed might also increase agricultural incomes in (rural) Uganda. In addition, a more globalised foodshed may strengthen international trade relations, which may be crucial in the future as climate change impacts might inhibit some regions to be food self-sufficient^62^. The present study indicates that established, high-income urban inhabitants, who have the most local consumption foodshed due to being active in urban agricultural activities, have lower levels of severe food insecurity, and enjoy a more diverse diet (Supplementary Table 2). However, the nutritional quality of the food grown in and around Kampala should be assessed as, in some cases, local food can be inferior to non-local food^63^. More research is required to assess how these future scenarios will affect Uganda’s food self-sufficiency and, in turn, the food security levels of its (urban) inhabitants, either negatively or positively. Therefore, to enable city managers to monitor these changes over time, it is crucial to quantify a baseline foodshed and, accordingly, to use the same foodshed boundaries in future visualisations^24^.

### Policy implications

These findings have urban and regional policy and planning implications. The foodsheds presented in this paper offer a quantitative and visual depiction of the flow of food into the city, providing valuable information for future land use planning and policy decisions that support economic development. Policy interventions need to be targeted towards the entire food system. The foodshed approach depicts the critical food production regions for the city. When combined with potential threats to food production in those regions through climate change or land use change, the threats to the food system can be assessed in more detail and interventions targeted to the critical regions.

Regarding (peri-) urban agriculture, the present analysis indicates that urban residents, and mainly those that are well-established in the city, heavily rely on this to access food. If urban managers wish to limit the extent of urban sprawl, ‘infilling’ scenarios^7^ focusing on densification of the built-up area will cause small-scale urban agricultural plots to disappear. On the other hand, a business-as-usual scenario of urban growth will cause for a more fragmented peri-urban agricultural landscape. Currently, much of the land ownership is unregulated in Kampala which results in agricultural land encroachments. For urban agriculture to thrive in the city, there is a need for well-framed land tenure policies which protect agricultural land^64^, and in doing so, manage the rural-urban interface. At the same time, to guarantee access to farming among all SECs in Kampala, there should be policies targeted at training, market inclusion and land provision. KCCA is currently setting up training centres teaching urban farmers how to sustainably grow food on a limited space e.g. using vertical gardens^65^.

Urban planning tends to be geographically targeted towards smaller areas. The present study unveils the need for a national-level spatial planning framework to ensure appropriate management of the foodshed of urban consumers. While mapping the city foodshed from a consumer perspective sheds light on urban food demand, it also uncovers the potential contribution of the urban food system to agricultural development and poverty alleviation in rural areas^24,25^. A focus on improving infrastructure in secondary cities could alleviate pressure on the rapid growth of Kampala and allow for more sustainable urban food systems transformations^17,43,53^. In short, detailed knowledge on the shape and size of the foodshed can aid urban planners in their strategy development to either decrease, preserve or increase the share of food sourced locally, within or near the GKMA. Future research on land use changes and/or food systems in Uganda should consider the current foodshed in their analysis.

### Limitations

Surveyed households were asked about the five main staple food items consumed by their household, which was deliberately left up to interpretation of the respondent. It is therefore possible they answered this question in terms of caloric intake, spending, weight or personal preference. Despite this limitation, our dataset includes a total of 3490 records or a mean of 4.67 staple foodstuffs per surveyed household, with a total combined foodshed value of 13,111,759 UGX/day or an equivalent of 3774 UGX/person/day for staple foods. This value spent on the five main staple foods relates closely to the average daily food expenditure per person in our dataset of 3781 UGX (Supplementary Table 1), which was included as a separate question in our survey. Moreover, this value is in the same order of magnitude as the average consumption expenditure (including food) of 4451 UGX/person/day in Kampala according to the 2012/2013 UBOS census^66^. Furthermore, the staple food groups mentioned by surveyed households closely match the overall pattern of the food groups sold by surveyed vendors (Supplementary Table 2). This validates our approach of focusing on the five main foodstuffs for each household. However, it should be noted that the foodshed quantities as mapped in this study should be interpreted as estimates rather than as an accurate measure of supply chain values. This is the case for most empirical foodshed studies, especially when including urban agriculture^24^.

While quantities of incoming food flows are based on the quantities indicated by surveyed consumers, our foodshed location origin data relies strongly on the accuracy with which food vendors were able to recall where the food they sold was grown. We selected this method due to the close interpersonal relationships market vendors foster with their suppliers, which is why they generally have an excellent understanding of where the food they sell was grown. Although there was a need for primary data collection, to our knowledge, our approach is currently the only study that starts from the consumer’s perspective and empirically traces food back to its origin, whether that is local (urban agriculture), regional or international. In addition, Supplementary Fig. 2 shows the results of a sensitivity analysis that was carried out using the K-folds cross validation method using *k* = 10 folds (see ‘Methods’ section). All but one of the test folds had a root mean squared error (RMSE) on their cumulative foodshed curve under 6.5% compared to its counterpart training dataset. The average RMSE across all data folds was 5.9%, which is satisfactory for the purposes of this study.

Another limitation is that we did not aim to quantify the role of the urban food system in food redistribution towards other areas (as per e.g.,^2^), since we consider the consuming households in Kampala to be the ‘end point’ of the mapped food flows. It is likely that supermarkets and wholesalers play an important role in the redistribution of food towards peri-urban or rural areas. Our limited sample size of *n* = 3 supermarkets and *n* = 3 wholesalers reflects the relatively low importance of these retail outlets in Kampala’s urban food system (in terms of household food purchasing). Further research could assess the role of markets in Kampala as redistribution centres towards peri-urban or rural areas, or even distant international markets. In addition, because our focus is on the city of Kampala, we have no comparative data on secondary cities in Uganda, where the share of urban agriculture might be even more substantial to the consumer foodshed due to lower housing densities^67^. Future studies could compare the dependency on (peri-) urban agriculture between cities.

### General discussion and outlook

The disentangled foodshed of consumers of various socio-economic clusters (SECs) in Kampala demonstrates that high- to middle-income urban dwellers adopt the tactic of diversifying the sources where they obtain food. Despite these tactics, the size of the foodshed does not differ significantly between dwellers of different SECs. We confirm the pattern observed at national level, that Uganda is highly self-sufficient, as we demonstrate that the food consumed by citizens in the capital city is also originating from within national boundaries for over 95%. Urban agriculture, sometimes considered an insignificant activity^12^, provides twice the equivalent retail value in food to consumers in Kampala as compared to international imports. While city dwellers are much less active in farming than their rural counterparts, urban agriculture is not to be dismissed when it comes to planning urban food strategies. Continued rapid urbanisation will therefore imply that changes in the urban food system will be different for each SEC. While the established high-income households are most at risk of losing (peri-) urban agricultural land to urban sprawl, they are also the group that is the most food secure and generally has access to the financial means to mitigate this risk. The low-income newcomers, who are the most food insecure, are the most vulnerable when it comes to changes in the food supply system of Kampala. They depend highly on retailers and lack the economic power to diversify their food sources. Established households with a low income face similar vulnerabilities as new rural-urban migrants with a middle income.

Many urban food systems analyses focus on production loss, changing diets or accessibility issues, but these food systems are rarely assessed spatially. When the geography changes rapidly due to urbanisation, globalisation, geopolitical conflict and climate change, mapping the foodshed can be an important tool in assessing the sustainability of the urban food system^25^. Spatial analyses of urban food systems often do not include the consumer’s viewpoint and generally consider the metropolitan area to be a homogenous consumer centre rather than a socially heterogeneous patchwork of potential consumers. In addition, urban agriculture is often disregarded in foodshed analyses. The present study includes these three elements. While scholars often advocate for highly localised food systems, especially in developing nations, in order to *“avoid the whims of international markets”*^28^ (Halweil, 2002, p.8), our study demonstrates that Uganda’s high self-sufficiency does not necessarily result in higher levels of food security for its urban inhabitants. More academic and policy attention needs to be directed towards understanding and quantifying urban foodsheds in other SSA cities.

## Methods

### Data collection

In order to map urban foodsheds, information from both food vendors and consumers (i.e., households) is required. We surveyed a total of 763 households and 305 food vendors in 25 parishes (the smallest administrative unit) of the Greater Kampala Metropolitan Area (GKMA). We surveyed 247 households and 118 food vendors in July-August 2019, 294 households and 82 food vendors in November-December 2019, and an additional 222 households and 105 food vendors in March-April 2021. The surveys were carried out by a total of nine different surveyors, using the Open Data Kit (ODK) Collection app (version 1.24.1).

### Sampling strategy

We used the smallest administrative unit (parish) to sample households and food vendors in locations that are contrasting in terms of their socio-economic population dynamics, as well as in terms of their geographical location within the GKMA. To select the 25 sampled parishes, we relied on the expertise of contributors at the Urban Action Lab at Makerere University combined with prior parish-level research on the socio-economic population dynamics^41^. Moreover, we considered practical accessibility and surveyor safety. Within the sampled parishes, a snowball strategy was adopted to select households for participation. A local council representative, after being explained the purposes of the research and giving their informed consent, led the surveyors to households and assisted surveyors to clarify the purpose of the study to ensure household informed consent. As such, a convenience sampling method was adopted on the field. To account for seasonality issues, we spread the household and vendor surveys out in time during both dry and rainy seasons. To sample food vendors, we aimed to survey the food vendors most frequently mentioned by the surveyed households regarding their food accessibility, to ensure that the staple food items could accurately be traced back to their origin location.

### Survey protocol

The household survey protocol consisted of five parts: household demographics, socio-economic characteristics, food accessibility (Supplementary Table 3), dietary diversity and food security. We calculated the Household Dietary Diversity Score^68^ and the Food Insecurity Experience Scale^48^ according to the methodologies defined by the Food and Agriculture Organisation of the United Nations (FAO). The food vendor survey consisted of three parts: vendor demographics, business strategies and food stock (i.e., their supply, Supplementary Table 4). The survey protocol was approved by the KU Leuven Social and Societal Ethics Committee (SMEC) on 19 June 2019 (approval no. G-2019 06 1664).

### Data processing

Data were initially uploaded to Google Spreadsheets, and downloaded in CSV format for further processing using Python (v. 3.9.7). The 541 households that were surveyed in 2019 were subjected to a missing data analysis. Households who did not provide an answer to over 37% of survey questions were excluded from analysis^46^. This way, 16 households who were sampled in 2019 were excluded from analysis. The remaining 525 households were clustered based on 72 socio-economic variables using a k-prototypes method in R (v. 4.2.0)^46,69^. The 222 households that were added to the dataset in 2021 were clustered separately using the same methodology. No household data collected in 2021 were excluded. Two surveyed food vendors were excluded from analysis as the vendor type was not documented. Thus, the final dataset used in this study contains *n* = 747 households and *n* = 303 food vendors.

Processing and cleaning the raw data on food consumption included the standardisation of units, creating a separate data file for vendor stock and household consumption, and converting all provided units to Ugandan shillings (UGX) per person per day. We did this by generating the average selling price in UGX per foodstuff unit (e.g., kilogram, piece, bunch, litre, cup, bucket, sack) indicated by food vendors. For example, ‘5 kgs of matoke per week’ for a family of 6 would convert to 193.5 UGX/person/day considering the average price of 1 kg of matoke as sold by the surveyed vendors was 1625 UGX across seasons. We allowed for survey respondents to indicate quantities in terms of kilograms, grams, litres, Ugandan shillings (UGX), pieces (e.g., ‘one pineapple’), buckets, cups, sacks, over a time period of days, weeks (7 days), or months (30.5 days). This required data cleaning and harmonisation. 0.60% of the household food consumption data was not converted to UGX/person/day, while for the vendors 5.57% of the supply data was not able to be converted to UGX/day. This is because missing household consumption data (households who mentioned a product but did not provide a unit) were replaced by the average daily retail value equivalent per person (UGX/person/day) for that product, while we were unable to do this for vendors as they vary strongly in business size and strategy.

### Mapping urban foodsheds

To map the foodshed of Kampala’s consumers by SEC, we trace the staple foods indicated by the surveyed households back to their origin. We asked households about the five food items they consume the most, what amount they usually consume, as well as how (from which vendor type) and where (what parish in Kampala) they obtained it. Our dataset of staple foodstuffs consumed by households contains *n* = 3490 items. Missing household consumption data, i.e., households who mentioned a staple food product but did not provide an amount or unit, were replaced by the average dataset UGX/person/day for that food item. If they produce the food themselves or if it was produced by family, friends or neighbours we asked them where the food was grown.

In the next step, we look for a food vendor in our dataset of the vendor type indicated by the household, in the location specified by the household, that sells the staple food item mentioned by the household. If no vendor is found within the location, we drop the location requirement and we use any vendor of that type. If there is still no data available for a vendor of that retailer type selling the staple food, we drop the vendor type requirement as well, i.e., we look for any vendor that sells this staple food item. Our dataset of foodstuffs sold by vendors in Kampala contains *n* = 1001 items (85 unique food types). The staple food is only excluded from analysis if no vendor information can be found for this staple food. This way, the following staple food items mentioned by household consumers were excluded: ‘Tea’ (*n* = 2), ‘Rolex’ (a typical Ugandan dish, *n* = 2), ‘Coffee’ (*n* = 1), ‘Ethiopian food’ (*n* = 1), ‘Grasshoppers’ (*n* = 1), ‘Cheese’ (*n* = 1), ‘Yoghurt’ (*n* = 1), ‘Fries’ (*n* = 1), and ‘Pizza’ (*n* = 1). As we focused on whole foods, it is expected that most of the excluded food items are processed dishes.

When the origin locations were found, a list of all parishes of the GKMA, a list of all districts of Uganda and a list of all other countries mentioned as food origins were filled with the equivalent retail value in UGX/person/day. These amounts were summed for each parish, district and country and added to an OpenStreetMap shapefile to visualise the foodshed in ArcGIS (v. 10.7.1). Similar to the foodshed mapping methodology proven suitable by Karg et al.^24^, we map the foodshed by delineating the smallest area that contains all the areas that contribute to the foodshed for 25%, 50%, 70% and 95%. We moreover plot the percentage of the foodshed against the distance from Kampala for each SEC, as well as for the food sold by each vendor type and each food group. The graphs produced for the vendor types and FAO-based food groups^68^ are based solely on the locations mentioned in our food vendor surveys, and do not depend on household consumption.

### Validation

A K-folds cross validation was carried out using Python (v. 3.9.7) where the household food consumption data (*n* = 3490) was split into *k* = 10 folds of *n* = 349 random observations each using scikit-learn^70^. The foodshed graph was calculated for each fold of test data, as well as for each counterpart set of training data (*n* = 3141 observations each). To assess the foodshed analysis accuracy, the average value of the root mean squared error (RMSE) for each pair of training and test data was calculated.

## Supplementary information

Supplementary Information

## Data Availability

The data supporting the findings of this study, collected using the survey protocol in Supplementary Tables 3 and 4, are protected by the KU Leuven Social and Societal Ethics Committee (SMEC) approval (no. G-2019 06 1664). Participants of this study did not agree for their data to be shared publicly. The anonymised, aggregated results dataset is available from the corresponding author upon reasonable request.

## Code availability

The Python code used for converting units, and tracing staple foods back to its origin, is available from the corresponding author upon reasonable request.
