# Supplementary material for: Mapping the consumer foodshed of the Kampala city region shows the importance of urban agriculture
Source: NPJ Urban Sustain. 2023 Mar 2;3(1):11. doi: 10.1038/s42949-023-00093-1 (PMC9978292; doi:10.1038/s42949-023-00093-1)
Supplement: Supplementary file 1 — Supplementary Information [file 42949_2023_93_MOESM1_ESM.pdf]

Supplementary Information for

Mapping the consumer foodshed of the Kampala city region shows the importance of urban agriculture

Lisa-Marie Hemerijckx[1,2,✉], Gloria Nsangi Nakyagaba[3,4], Hakimu Sseviiri[3], Katarzyna Janusz[1], Michelle Eichinger[5], Shuaib Lwasa[3,6], Julian May[7], Peter H. Verburg[5], Anton Van Rompaey[1]

[1] Department of Earth and Environmental Sciences, KU Leuven, Belgium
[2] Fonds Wetenschappelijk Onderzoek (FWO) Vlaanderen, Belgium
[3] Urban Action Lab (UAL), Department of Geography, Geo-Informatics and Climatic Sciences, Makerere University, Uganda
[4] Department of Geography and Environmental Sustainability, University of Oklahoma, United States of America
[5] Institute for Environmental Studies (IVM), Vrije Universiteit Amsterdam, The Netherlands
[6] International Institute of Social Studies (ISS), Erasmus University Rotterdam, The Netherlands
[7] DSI-NRF Centre of Excellence in Food Security, University of the Western Cape, South Africa

✉ corresponding author, e-mail: lisamarie.hemerijckx@kuleuven.be

Supplementary results

Supplementary Table 1 | Summary of characteristics defining the four socio-economic clusters used in this study [1–4]. M = mean, Mdn = median, SD = standard deviation.

Characteristic	Established high income n = 221	Established low income n = 134	Newcomers middle income n = 177	Newcomers low income n = 215	Dataset (households) n = 747
**Monthly income per person (UGX)**	M = 376,643<br>Mdn = 181,250<br>SD = 640,881	M = 99,223<br>Mdn = 75,000<br>SD =93,790	M =156,897<br>Mdn = 125,000<br>SD = 136,873	M = 105,287<br>Mdn = 75,000<br>SD = 91,824	M = 201,125<br>Mdn = 112,500<br>SD = 387,819
**Daily food expenditure per person (UGX)**	M = 4,618<br>Mdn = 2,779<br>SD = 7,002	M = 2,856<br>Mdn = 2,500<br>SD = 2,077	M = 4,226<br>Mdn = 3,000<br>SD = 4,419	M = 3,121<br>Mdn = 2,500<br>SD = 3,755	M = 3,781<br>Mdn = 2,500<br>SD = 4,949
**Household size**	M = 6.23<br>Mdn = 6<br>SD = 3.63	M = 5.25<br>Mdn = 5<br>SD = 2.80	M = 4.31<br>Mdn = 4<br>SD = 2.62	M = 4.46<br>Mdn = 4<br>SD = 2.43	M = 5.09<br>Mdn = 5<br>SD = 3.04
**Urban agricultural activity**	65.16%	60.45%	21.47%	24.65%	42.30%
**Years lived in Kampala**	M = 23.81<br>Mdn = 21<br>SD = 18.97	M = 24.98<br>Mdn = 20<br>SD = 21.37	M = 11.63<br>Mdn = 4.25<br>SD = 20.56	M = 14.37<br>Mdn = 6<br>SD = 17.58	M = 18.43<br>Mdn = 10<br>SD = 20.21
**Severe food insecurity prevalence (FAO Food Insecurity Experience Scale)**	4.82%	17.08%	12.54%	21.85%	13.43%
**Household Dietary Diversity Score (FAO 12 food groups)**	M = 6.35<br>Mdn = 6<br>SD = 2.00	M = 5.42<br>Mdn = 5.5<br>SD = 1.68	M = 6.10<br>Mdn = 6<br>SD = 1.66	M = 5.14<br>Mdn = 5<br>SD = 1.89	M = 5.77<br>Mdn = 6<br>SD = 1.90
**Location in the study area**	Mainly on large homes on hilltops in the inner city or on the outskirts of the GKMA	Spread relatively evenly across GKMA	Spread relatively evenly across GKMA	Mainly small homes or slum dwellings in the inner city	Greater Kampala Metropolitan Area (GKMA)

Supplementary Table 2 | Food groups [4] consumed as staple foods by surveyed households (n=747) and sold by food vendors (n=303) in Kampala. Percentage in terms of UGX/day spent on each food group.

Food group	Established high income n = 221	Established low income n = 134	Newcomers middle income n = 177	Newcomers low income n = 215	Dataset (households) n = 747	Mobile street vendor n = 16	Fixed street vendor n = 112	Market vendor n = 112	Retailer n = 57	Super-market n = 3	Wholesaler n = 3	Dataset (vendors) n = 303
Cereals	18.81	24.64	28.62	52.09	31.73	0.00	0.96	0.34	46.97	51.73	40.10	8.43
White roots and tubers	28.36	30.34	22.07	17.31	24.14	9.78	19.42	40.81	6.84	0.00	0.00	27.02
Vitamin A rich vegetables and tubers	0.05	0.00	0.09	0.11	0.07	2.69	0.39	0.10	0.02	0.00	0.00	0.24
Dark green leafy vegetables	1.41	1.07	2.22	0.50	1.22	0.87	0.94	0.84	0.15	0.00	0.00	0.75
Other vegetables	0.37	0.63	0.56	0.49	0.48	4.56	1.40	1.13	0.53	0.00	0.00	1.17
Vitamin A rich fruits	0.47	0.00	0.00	0.00	0.17	1.41	1.54	1.37	0.01	0.00	0.00	1.20
Other fruits	0.25	0.15	0.46	0.08	0.22	41.53	3.68	4.23	1.09	0.00	0.00	4.30
Organ meat	0.16	0.36	0.00	0.09	0.14	0.00	4.79	1.92	0.00	0.00	0.00	2.50
Flesh meats	27.41	17.68	22.46	10.15	19.67	0.00	49.38	36.15	0.00	0.00	0.00	33.53
Eggs	1.16	0.06	0.09	0.10	0.46	0.00	0.33	0.03	1.52	0.00	0.05	0.34
Fish and seafood	7.86	8.57	8.90	6.38	7.71	31.57	7.82	10.92	0.00	0.00	0.00	8.48
Legumes, nuts and seeds	12.89	16.20	13.86	12.47	13.46	5.77	2.93	2.00	20.34	6.84	49.34	6.03
Milk and milk products	0.67	0.29	0.33	0.23	0.41	0.00	6.12	0.10	15.03	23.28	0.00	4.40
Oils and fats	0.00	0.00	0.00	0.00	0.00	0.00	0.19	0.01	0.06	4.51	0.00	0.11
Sweets	0.09	0.00	0.04	0.00	0.04	1.80	0.02	0.02	2.92	10.20	10.52	0.78
Spices, condiments and beverages	0.04	0.00	0.31	0.01	0.07	0.03	0.10	0.03	4.52	3.44	0.00	0.72

Supplementary Figure 1 | Foodshed maps per socio-economic cluster. Polygons delineate the percentage of food that is provisioned within that area. The dots represent district share of the SEC foodshed (in terms of UGX/day). The dominant food group originating from each district (based on household consumption) is also shown. Food groups not shown in the legend were not dominant in any district shown on the map extent.

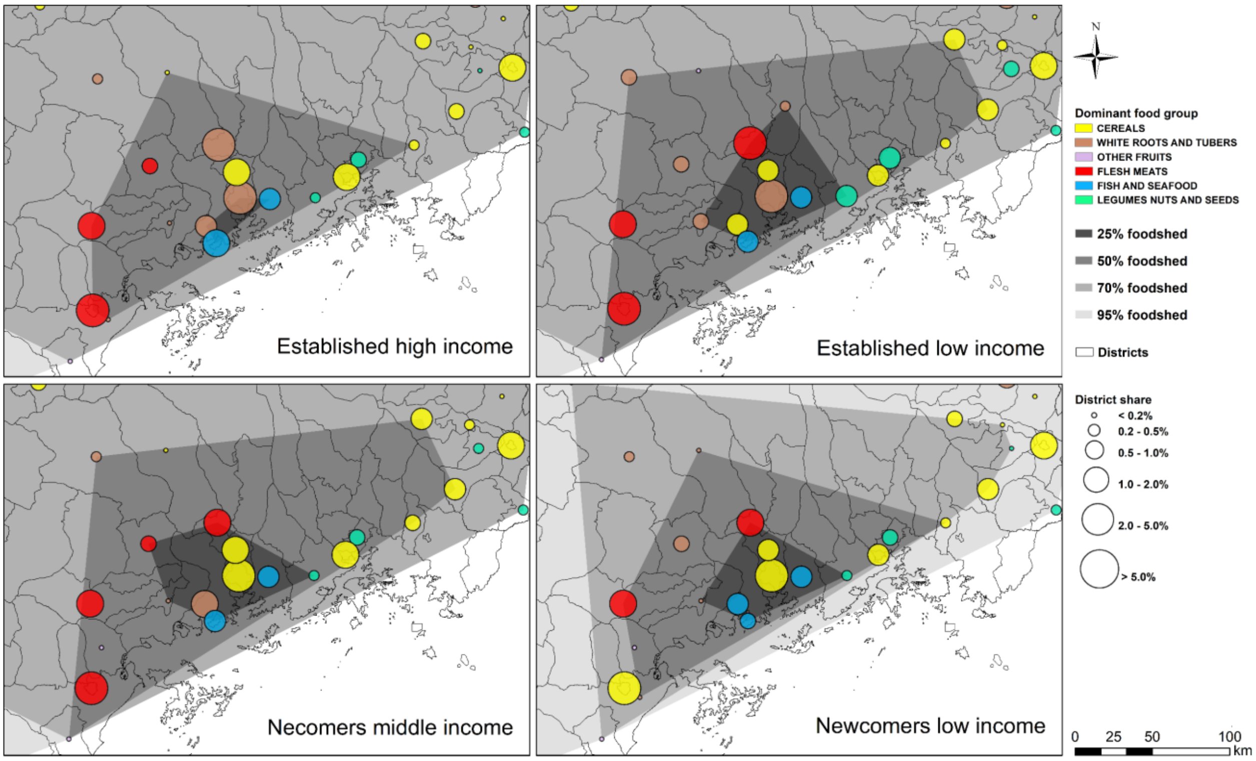

Supplementary Figure 2 | Sensitivity analysis: K-folds cross validation. The household food consumption data was split evenly into k=10 random, mutually exclusive folds (each containing 10% of the original dataset). The graph on the left demonstrates the cumulative contribution to the foodshed by distance to Kampala for each randomised fold. The graph on the right shows the root mean squared error (RMSE) for each fold, as well as the average RMSE for all data folds.

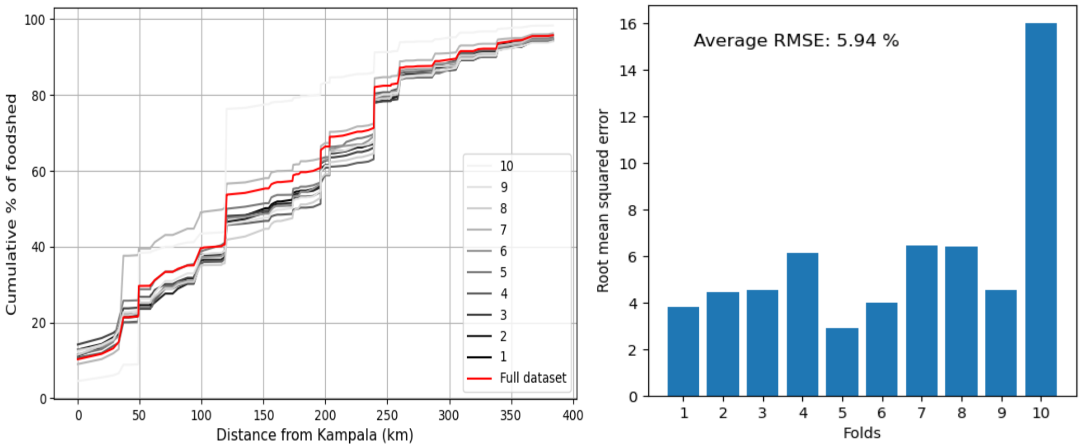

Supplementary Methods

Supplementary Table 3 | Survey protocol for household food access.

Variable	Survey Question	Input type
**Staple food name**	Can you describe the 5 main staple food items that your household consumes?	Free text (e.g. "Rice")
**For each staple food item mentioned, the questions below are asked:**		
**Quantity**	When you purchase this food, how much of it do you buy at once?	Number value
**Unit**	Surveyor: What unit did the respondent express the [Staple food name] in?	Ugandan shillings (UGX); Kilograms (kg); grams (g); litres (L); pieces (unit); bucket; cup; sack; bunch
**Frequency**	Surveyor: Did the respondent express this quantity per day or per week?	Day; Week
**Obtained where**	Where is the food obtained?	Home grown; Family or friends; Neighbours; Mobile street vendor; Fixed street vendor; Market vendor; Retailer; Supermarket
**Location**	Please provide the name and location of where you obtained the food.	Free text (e.g. "Bukoto market vendor" or "Home grown in back yard")
**Reason**	What is the main reason you bought or obtained the food at this specific location?	I know this vendor/source personally (trust); This is the cheapest way to obtain this food; It is close by; They have healthy options; They have good quality products; They offer a large and/or unique range of products
**Distance**	How long (travel time) does it take you to get to the [Location]?	Less than 15 minutes; 15-30 minutes; 31-60 minutes; 61-90 minutes; More than 90 minutes
**Transport**	What transport do you use to go to [Location]?	From home; Walking; Bicycle; Bodaboda; Taxi(bus); Own motorcycle; Own car/van; Company vehicle

Supplementary Table 4 | Survey protocol for vendor food access.

Variable	Survey Question	Input type
**Product name**	Can you name up to 10 most important food products that you sell?	Free text (e.g. "Rice")
**For each food item mentioned, the questions below are asked:**		
**Quantity**	How much [Product name] do you usually buy at once?	Number value
**Unit**	Surveyor: What unit did the respondent express the [Product name] in?	Kilograms (kg); grams (g); litres (L); pieces (unit); bucket; cup; sack; bunch
**Frequency**	How often do you obtain this [Quantity] [Unit] of [Product name]?	Every n days, n=0.5 (twice or more per day); n=1 (once a day); n=3 (a few times a week); n=7 (one a week); n=14 (a few times a month); n=120 (a few times a year); n=365 (yearly or rarely)
**Obtained where**	Where is the food obtained?	Home grown; Family or friends; Neighbours; Mobile street vendor; Fixed street vendor; Market vendor; Retailer; Supermarket
**Retail value**	At what price (UGX) do you sell [Product name] to your customers?	Number value (UGX)
**Price unit**	Surveyor: What unit did the respondent express the [Retail value] in?	Kilograms (kg); grams (g); litres (L); pieces (unit); bucket; cup; sack; bunch
**Supplier type**	What is the supplier type for this product?	Vendor grows this product themselves;<br>Gifted by a household that grows this themselves;<br>Directly from the farm; Middlemen;<br>Product obtained at another food market;<br>Reselling food from a retailer
**Origin**	What is the (main) origin of the [Product name]? You may indicate more than one location.	Origin types: Kampala; Uganda; Abroad
	IF Origin is Kampala: What parish in Kampala was this product grown in?	Selection from list of 181 parishes in the Greater Kampala Metropolitan Area
	IF Origin is Uganda: What district(s) in Uganda was this product grown in?	Selection from list of 135 districts in Uganda
	IF Origin is Abroad: What country was this product imported from?	Free text (e.g. "Tanzania")

Supplementary references

1. Hemerijckx, L.-M. *et al.* Upscaling household survey data using remote sensing to map socioeconomic groups in Kampala, Uganda. *Remote Sens.* **12**, 3468 (2020).
2. Hemerijckx, L.-M. *et al.* Food accessibility of different socioeconomic groups in sub-Saharan African cities: a mixed-method analysis in Kampala, Uganda. *Food Secur.* **14**, 677–694 (2022).
3. Cafiero, C., Viviani, S. & Nord, M. Food security measurement in a global context: The food insecurity experience scale. *Measurement* **116**, 146–152 (2018).
4. Kennedy, G., Ballard, T. & Dop, M. C. *Guidelines for measuring household and individual dietary diversity*. (FAO, 2010).
